# LED-supplied blue light mitigates ammonium toxicity in rapeseed (*Brassica napus* L.)

**DOI:** 10.3389/fpls.2025.1724504

**Published:** 2025-12-16

**Authors:** Wenjing Li, Jinnan Song, Qingbing Sun, Jingli Yang, Jingmin Zhang, Haicheng Xu, Dianliang Peng, Maopeng Sang, Byoung Ryong Jeong

**Affiliations:** 1 Shandong Provincial University Laboratory for Protected Horticulture, Weifang University of Science and Technology, Shouguang, China; 2 Division of Horticultural Science, College of Agriculture and Life Sciences, Gyeongsang National University, Jinju, Republic of Korea; 3 Jingzhi-Maoteng Agricultural Technology Limited Company in Qushui County, Lhasa, Tibet, China

**Keywords:** antioxidant enzymes, nitrogen assimilation pathway, nitrogen nutrition, photosynthetic ability, rapeseed

## Abstract

Ammonium (NH_4_^+^) toxicity adversely curtails the growth and productivity of rapeseed plants. Current knowledge shows that blue (B) light is an alternative approach used to minimize or alleviate disturbances caused by various abiotic stresses. However, few studies have investigated NH_4_^+^-stressed rapeseed plants to illustrate the alleviatory role of blue light. Therefore, this study was conducted to determine whether blue light could reduce the degree of NH_4_^+^ toxicity in rapeseed and, at the same time, elucidate the underlying mechanism. To this end, rapeseed plants were cultured in a controlled environment (14 h light at 22°C and 10 h dark at 18°C) and treated with one of three NH_4_^+^:NO_3_^−^ regimes (0:100, 50:50, and 100:0) with a constant nitrogen concentration of 13 me L^−1^, under white light-emitting diode (LED) light or blue LED light at 200 PPFD. Plants treated exclusively with NH_4_^+^ under white light exhibited decreased growth, disturbed photosynthesis, inhibited antioxidant defense systems, limited nitrogen (N) assimilation, and ultimately developed NH_4_^+^ toxicity symptoms (as characterized by chlorosis, necrosis, and stunted morphology). These traits and parameters were significantly mitigated by blue light treatment. Collectively, this study highlights the benefits of blue light on plants, particularly for NH_4_^+^-sensitive species such as rapeseed.

## Graphical Abstract

**Figure:**
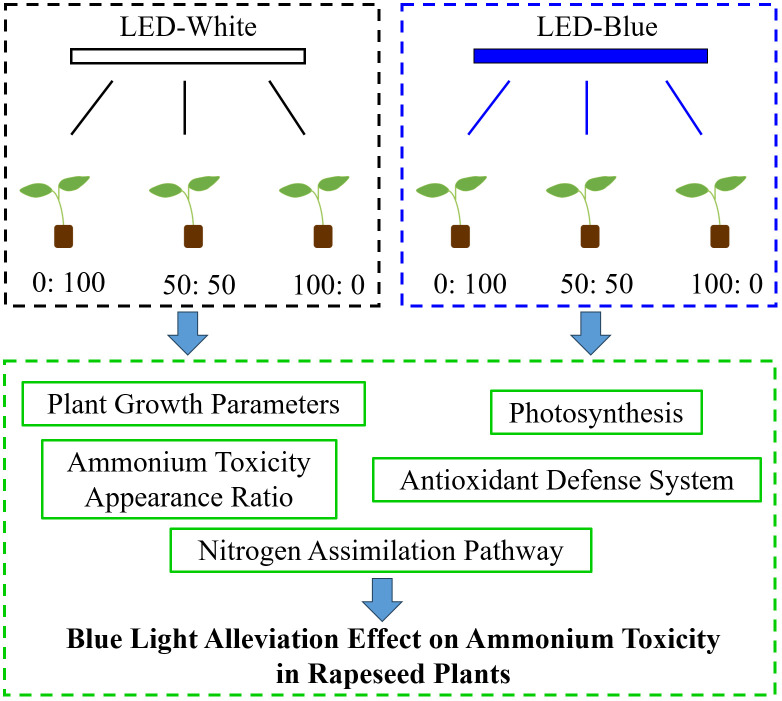

## 1 Introduction

Nitrogen (N) nutrition is of paramount importance for plant structural development and productivity, as it comprises many organic compounds, such as the amino acids, nucleic acids, and proteins (Bloom, 2015; Leghari et al., 2016). When limited, it severely reduces plant biomass. This fundamental element is absorbed mainly in forms of nitrate (NO_3_^−^) and ammonium (NH_4_^+^) (Song et al., 2022a). The availability, uptake, and metabolism of these two inorganic N sources by plants were found to differ markedly in their energetic, biological, and biochemical processes (Luo et al., 2013; Guo et al., 2007). Theoretically, the NH_4_^+^ can be readily assimilated by plants, whereas NO_3_^−^ is energy-consuming (Bittsánszky et al., 2015). NH_4_^+^ is also less prone to leaching, conferring higher assimilation efficiency and lower environmental pollution. Additionally, many edible leafy vegetables were more prone to accumulate nitrite (NO_2_^−^), an intermediate product in the nitrogen assimilation pathway that is regarded as toxic to both human health and plant growth (Picetti et al., 2022).

Paradoxically, exclusively or unintentionally applied NH_4_^+^ nutrition can result in plant tissue acidification (Hachiya et al., 2021), and the plants ultimately manifest ammonium toxicity (or NH_4_^+^ toxicity). This phenomenon has been observed in many NH_4_^+^-sensitive plant species, such as cucumber (Roosta and Schjoerring, 2007), cabbage (Song et al., 2022a), salvia (Song et al., 2022b), and basil (Song et al., 2024a). During this period, several morphological and physiological dysfunctions can occur due to interrupted metabolism. Typically, plants exhibiting NH_4_^+^ toxicity show visual detrimental signs, including reduced growth, leaf chlorosis and necrosis, and stunted roots (Esteban et al., 2016). Meanwhile, certain integrated internal impacts may also be elicited: photosynthesis can be disturbed (Wang et al., 2019; Bittsánszky et al., 2015), oxidative stress in terms of reactive oxygen species (ROS) and corresponding scavenging enzymes can be increased (Song et al., 2022b; Esteban et al., 2016), and major enzymes in the NH_4_^+^ assimilation pathway can be significantly altered (Song et al., 2021; Bittsánszky et al., 2015; Cruz et al., 2006; Song et al., 2022a). However, the NH_4_^+^ tolerance in plants can be induced by improving the expression of glutamine synthetase (GS) and glutamate dehydrogenase (GDH); additionally, Xian et al. (2020) suggested that increasing GDH activity is an important strategy for NH_4_^+^ detoxification (Song et al., 2021, 2022).

Rapeseed (*Brassica napus* L.), known as oilseed rape, is an important and prolific oil crop that is not only used as a high-quality edible oil for humans but also serves as feed for animals and as a source of lubricants (Zhang and Flottmann, 2016). Its agro-industrial value has promoted large-scale cultivation worldwide; for example, the production area in China has exceeded 7 million hectares since 2019 (Lei et al., 2019). However, its certain types or varieties are sensitive to high NH_4_^+^ nutrition (Zhou et al., 2024; Li S et al., 2024). As a result, the yield and quality of rapeseed are often limited by inappropriate application of NH_4_^+^-containing fertilizers. Moreover, overuse of NH_4_^+^ fertilizers results not only in low nitrogen use efficiency (NUE) but also in significant environmental hazards. Therefore, agronomic practices or other horticultural strategies to increase plant NH_4_^+^ tolerances are recommended. Recently, researchers have improved plant tolerance to abiotic stresses by adjusting the photoenvironment, since light is an essential environmental signal determining plant growth and development (Ma et al., 2022; Morello et al., 2022; Ren et al., 2023).

Light quality, optical intensity, photoperiod, and light distribution are the predominant factors that impart a plethora of physiological effects on plants (Yamori et al., 2020; Trojak et al., 2022). The specific wavelength of light exerts precise influences on the quantum yield basis of photosynthesis. In other words, different light qualities carrying varying levels of energy affect plants by regulating a variety of biological processes, such as tissue differentiation and nutrient absorption (Lee et al., 2021). In particular, blue (B) light is regarded as the most efficient spectrum for photosynthesis, steering photomorphogenesis, cell division, leaf expansion, stomata opening, and pigment accumulation (Naznin et al., 2019). It has been reported that photosynthetic capacity significantly improves when the percentage of blue light increases in a red-light background (Hogewoning et al., 2010). Moreover, blue light treatment has been suggested to reinforce antioxidant capacity (Zhang et al., 2023; Ren et al., 2023), regulate nitrogen metabolism (Ren et al., 2023), and suppress symptoms of multiple abiotic stresses (Ren et al., 2023; Roeber et al., 2021). Recently, light-emitting diodes (LEDs) have emerged as a spectrally and energetically optimal alternative for maximum crop production in plant factories with artificial lighting (PFALs). A great deal of reports have also shown that manipulating LED-supplied blue light is a sustainable and powerful method for altering plant traits under environmental stresses (Kong and Zheng, 2023). However, studies on whether blue light treatment can reduce NH_4_^+^ toxicity in rapeseed (*Brassica napus* L.) are very scarce (Liu et al., 2020; Li S et al., 2021, 2024).

Therefore, the current study was undertaken to (1) characterize NH_4_^+^ toxicity in rapeseed by applying a high level of NH_4_^+^, (2) determine whether blue light can mitigate NH_4_^+^ toxicity in rapeseed, and (3) ascertain the alleviatory effects of blue light on rapeseed growth attributes, photosynthetic capacity, antioxidant defense system, and nitrogen metabolism machinery.

## 2 Materials and methods

### 2.1 Plant material and culture conditions

Rapeseed seeds “Qinyou” were selected as the plant material and purchased from Ronghua Agriculture Technology Co. Ltd. (Xian, Shanxi, China). Full-grain seeds of uniform size and without any mechanical damage were selected and sown in 128-cell plug trays filled with mini-K medium (Klasmann–Deilmann GmbH Company, Geeste, Germany). After sowing, the medium was carefully moistened with tap water and covered with cling film to preserve moisture until germination.

**Figure 1:**
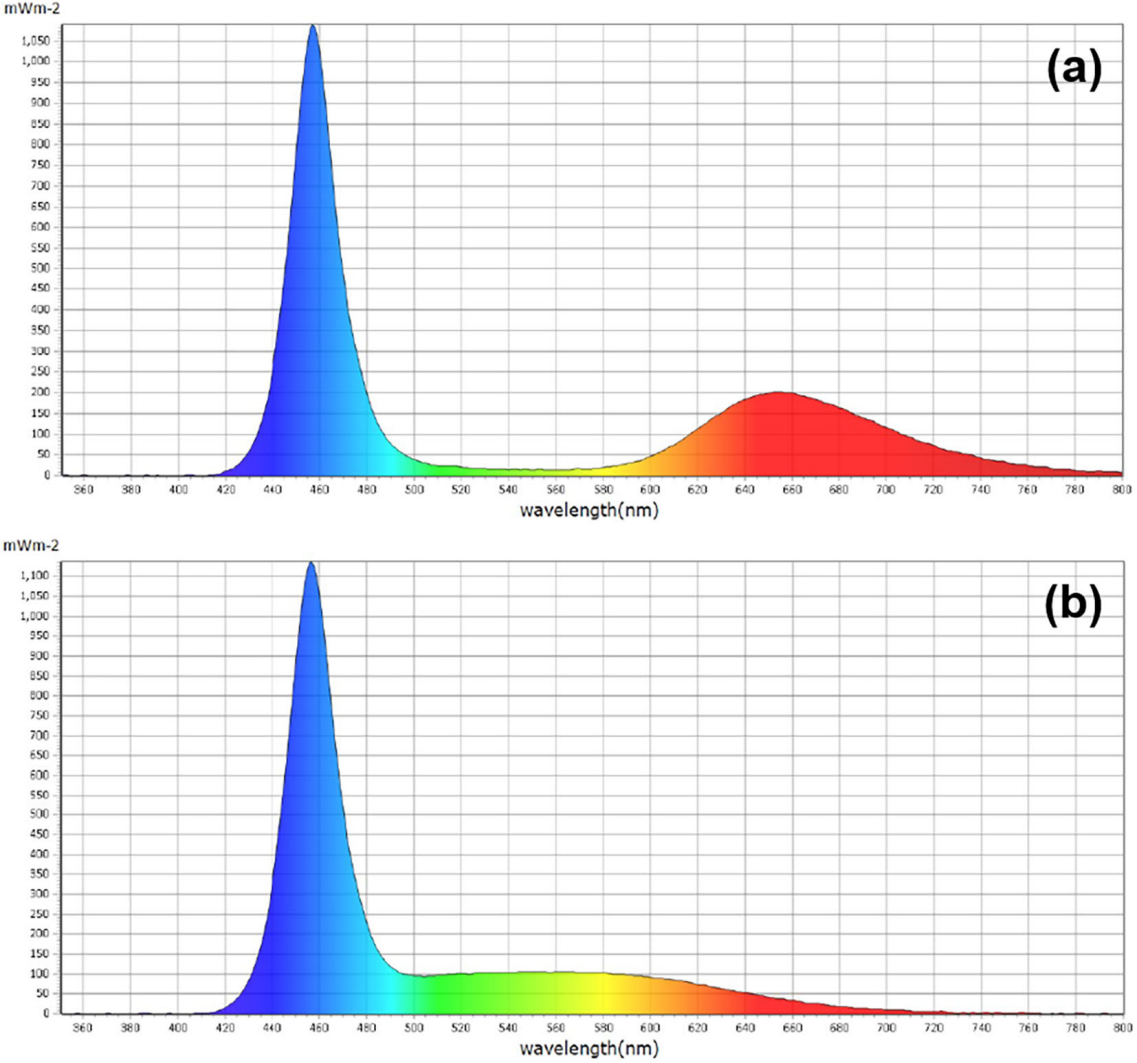
The spectral distributions of **(a)** white LED light and **(b)** blue LED light supplied for the culture of rapeseed.

Rapeseed seeds were germinated 7 days after sowing (DAS) under an air-conditioned environment at 22 °C ± 2°C in darkness. The germinated rapeseed seeds were quickly transferred to a controlled alternating diurnal regime (14 h light at 22°C and 10 h dark at 18°C) and irrigated with multipurpose nutrient solution (MNS) according to our previous publications (Song et al., 2022a, 2022). The light environment was measured with a handheld spectrometer (PG200N Spectral PAR Meter, UPRtek, Miaoli County, Taiwan). Light condition was provided by white or blue LED light with an intensity of 200 µmol m^−2^ s^−1^ PPFD. The peak and dominant wavelengths of white LED light adopted were at 456 and 650 nm, respectively, while the peak wavelength of blue LED light was 456 nm (**Figure 1**).

The rapeseed seedlings were allowed to develop for another 6 days until they entered the growing stage with two true leaves and one heart (13 DAS). They were then cultivated with distilled water for another 3 days to get all the nutrients leaching out (16 DAS). Healthy seedlings of uniform size and similar morphology, without mechanical flaws or disease, were screened, selected, and transplanted into a new 128-cell plug tray. The transplanted seedlings were simultaneously subjected to different treatments (17 DAS).

### 2.2 The experimental treatments and design

The treatment solutions were prepared with three different NH_4_^+^:NO_3_^−^ ratios (0:100, 50:50, and 100:0), designated according to MNS with a constant N concentration at 13 me L^−1^. The detailed recipe and sourced chemicals are listed in **Table 1**. Transplanted seedlings under the three NH_4_^+^:NO_3_^−^ regimes were equally divided into six parts and subjected to white LED light or blue LED light treatment (see the Graphical Abstract).

**Table 1:** The nutrient composition (me L^−1^) for the treatment solutions in this study.

Chemicals used	NH_4_^+^:NO_3_^−^		
	0:100	50:50	100:0
Ca(NO_3_)_2_·4H_2_O	6.9	5.9	–
KNO_3_	4.8	–	–
Mg(NO_3_)_2_·6H_2_O	1.3	0.6	–
MgSO_4_·7H_2_O	1.0	1.4	1.7
KH_2_PO_4_	1.0	–	2.0
NH_4_H_2_PO_4_	–	2.0	–
(NH_4_)_2_SO_4_	–	4.5	13.0
K_2_SO_4_	–	4.5	1.2
CaCl_2_·6H_2_O	–	–	4.9

Overall, the three NH_4_^+^:NO_3_^−^ ratios combined with two LED light qualities formed the experimental treatments in this study. The trial was arranged as a 2 × 3 factorial scheme in a completely randomized design, with three biological replicates. A total of 24 rapeseed seedlings were planted for one replicate per NH_4_^+^:NO_3_^−^ ratio in this experiment.

### 2.3 Determination of plant growth parameters and destructive sampling

Subsequently, rapeseed plants subjected to different treatments were harvested (32 DAS) when they reached contrasting statuses and morphologies. The plants were first removed from the substrates and washed with distilled water. Plant roots were then surface-blotted with absorbent paper. Whole plant fresh biomass and dry weight (air-forced oven at 70°C for 48 h) were determined using an electronic balance. Shoot length, leaf length and width, and tap root length were measured with a metal ruler. Stem diameter was determined with a vernier caliper (CD-20CPX, Mitutoyo Korea Co., Gunpo, South Korea). Root volume, root surface area, and total root length were measured using a root analysis Microtek ScanWizard Pro system (MICROTEK, Shanghai, China). Leaf samples from different treatments were individually collected, immersed in liquid N_2_, and stored at − 80°C until further analysis.

### 2.4 Assessment of plant photosynthetic ability

Photosynthetic ability in this study was assessed using major parameters, including the net photosynthesis rate (Pn), stomatal conductance (*g*_s_), transpiration rate (Tr), chlorophylls (chlorophyll *a* and *b*), and carotenoids.

Specifically, the first three traits were measured with a portable photosynthesis measurement system (TARGAS-1, PP Systems, Amesbury, MA, USA). The three topmost fully expanded leaves were used for measurement, and each leaf was measured three times. During measurement, the leaf temperature was about 22°C, and the environment was identical to that previously set for culturing rapeseed. Photosynthesis-related pigments, including chlorophyll *a*, chlorophyll *b*, and carotenoids, were determined following a procedure by Sims and Gamon (2002): The absorbance of the extraction buffer (45% v/v acetone, 45% v/v ethanol, and 10% v/v H_2_O) was read at 645, 663, and 440 nm using a spectrophotometer (UV5100, Metash Instruments Co. Ltd., Shanghai, China), and the content was calculated using the following equations:


$$\text{Chlorophyll }a=\frac{(12.72\times \text{OD}\;663–2.59\times \text{OD}\;645)\times \text{V}}{\;\text{Sample Fresh weight}}$$

$$\text{Chlorophyll }b=\frac{(22.88\times \text{OD}\;645–4.67\times \text{OD}\;663)\times \text{V}}{\text{Sample Fresh weight}}$$

$$\text{Carotenoids}=\frac{4.7\times \text{OD}\;440–0.27\times (\text{Chl}\;a+\text{Chl}\;b)}{\text{Sample Fresh weight}}$$

Here, “V” is 2 ml (the volume of the extraction buffer), and the chlorophyll content is expressed in milligrams per gram of leaf fresh weight (mg g^−1^ FW).

### 2.5 Calculations of the appearing ratio of ammonium toxicity (%)

Rapeseed plants developed ammonium toxicity symptoms in response to a 100% NH_4_^+^ supply. The appearance ratio of ammonium toxicity (%) per replicate was calculated using the following equation:


$$\begin{array}{l} \text{Appearing ratio of ammonium toxicity }(\%)\\ =\frac{\text{Number of plants displaying ammonium toxicity symptoms}}{24}\\ \times 100\% \end{array}$$

Where “24” represents the number of rapeseed plants per treatment per replicate.

### 2.6 Estimation of the antioxidant defense system

The antioxidant defense system was estimated in terms of the main antioxidant enzyme activities and ROS accumulation. The antioxidant enzymes mainly consisted of superoxide dismutase (SOD), ascorbate peroxidase (APX), catalase (CAT), guaiacol peroxidase (GPX), dehydroascorbate reductase (DHAR), and glutathione reductase (GR). The ROS level mainly included the superoxide (O_2_.^−^) and hydrogen peroxide (H_2_O_2_).

Specifically, about 100 mg of finely ground leaf powder samples were well mixed with an extraction buffer (1 mM EDTA, 50 mM PBS, 2% polyvinylpyrrolidone, and 0.05% Triton-X at pH 7.0). This mixture was centrifuged (12,000 rpm, 4°C, 20 min) to obtain the supernatant, which was subsequently used for the quantification of total protein content (Bradford, 1976) and antioxidant enzyme activities (Song et al., 2022b). SOD activity was determined based on the reduction of nitroblue tetrazolium (NBT) (Giannopolitis and Ries, 1977). APX activity was measured using a method based on ascorbate oxidation (Nakano and Asada, 1981). The decomposition of H_2_O_2_ was used to determine CAT concentration (Cakmak and Marschner, 1992). GPX activity was assessed using the guaiacol oxidation reaction (Amako et al., 1994). DHAR activity was measured following the approach proposed by Nakano and Asada (1981). GR activity was determined using a rapid and sensitive procedure described by Mavis and Stellwagen (1968). O_2_.^−^ content was measured based on hydroxylamine oxidation by Wu and von Tiedemann (2002). H_2_O_2_ levels were colorimetrically determined following the protocol by Mukherjee and Choudhuri (1983).

### 2.7 Determination of NO_3_^−^, NO_2_^−^, and NH_4_^+^ content

Salicylic acid nitration was used to measure NO_3_^−^ content according to Cataldo et al. (1975). A quick method based on the Griess reaction was adopted for the determination of NO_2_^−^ concentration (Moshage et al., 1995). A colorimetric method based on the Berthelot reaction was employed to measure NH_4_^+^ content (Bräutigam et al., 2007). The specific steps can be found in Huang’s report (Huang et al., 2018).

### 2.8 Analysis of the key enzyme activities in the N-assimilation pathway

The key enzymes in the N-assimilation pathway mainly include nitrate reductase (NR), nitrite reductase (NIR), GS, glutamate synthetase (GOGAT), and Nicotinamide adenine dinucleotide (NADH)-dependent GDH, which were spectrophotometrically assayed using a spectrophotometer (UV3200, OptoSky, Xiamen, China) following our previous publications with minor modifications (Song et al., 2021, 2022). The NR activity was measured *in vitro* in accordance with a sensitive method by Högberg et al. (1986) and was expressed by the amount of nitrite formed, while the NIR concentration was determined by the reduction of NO_2_^−^ during assay (Ogawa et al., 1999). Specifically, 0.5 g of finely ground leaf samples were homogenized in a 5-ml protein extraction medium containing Tris-HCl at 50 mM, MgSO_4_ at 2 mM, DTT-dithiothreitol at 2 mM, sucrose at 400 mM, and pH 8.0. This mixture was centrifuged (13,000 rpm, 4°C, 20 min) to obtain the supernatant, which was later used for the determination of the enzymes of GS, GOGAT, and NADH-GDH.

The GS activity was estimated following a method by Oaks et al. (1980): A total of 0.7 ml crude enzyme extract was mixed with 2.3 ml assay solution (0.1 M Tris-HCl, 80 mM Mg^2+^ and hydroxylamine hydrochloride, 2 mM EGTA, 20 mM sodium glutamate and cysteine, and 40 mM daily prepared ATP, pH 7.4) and was then subjected to incubation at 37°C for 30 min. To terminate the reaction, 1 ml of ferric chloride reagent (0.37 M FeCl_3_, 0.6 M HCl, and 0.2 M TCA) was added. The mixture was then vigorously shaken for 5 min and centrifuged (5,000×*g*, RT, 10 min) to obtain the supernatant, which was subsequently recorded spectrophotometrically at 540 nm. One unit of GS activity was defined as the synthesis of 1 μmol γ-glutamyl hydroxamate per hour per gram of fresh weight.

The GOGAT activity was determined based on an approach as presented by Lin and Kao (1996): 0.5 ml crude enzyme extract was added to a reaction medium (0.1 ml KCl at 10 mM, 0.2 ml NADH at 3 mM, 0.05 ml α-oxoglutarate at 0.1 M, and 0.4 ml l-glutamine at 20 mM, pH 7.6). The change of the absorbance of this mixture was spectrophotometrically read at 340 nm. The GOGAT activity was expressed as the change of absorbance at 0.001 per hour.

The NADH-GDH activity was assessed in accordance with a report by Kanamori et al. (1972): The reaction was triggered by adding 0.1 ml of crude enzyme extract to the 2.9-ml assay solution (distilled water at 0.3 ml, NH_4_Cl at 231 mM, α-ketoglutarate at 23.1 mM, Tris-HCl at 15.4 mM, 0.1 ml of CaCl_2_ at 30 mM, and 6 mM NADH). The absorbance of this mixture was immediately measured at 340 nm after a water bath at 37°C for 5 min. The NADH-GDH activity was characterized as the formation of nanomoles NAD^+^ per gram of fresh weight per minute.

### 2.9 Statistical analysis and graphs

The data displayed in this study were means ± SE from no less than three independent biological replicates (*n* ≥ 3). The statistical analysis of all data was performed with the SAS v8.0 program (SAS 8.2 Inst., Cary, NC, USA) by one-way analysis of variance (ANOVA) following Duncan’s multiple comparison range test at *p* = 0.05. The bar graphs were plotted using GraphPad Prism 8.0 software (GraphPad Software, Boston, MA, USA). The principal component analysis (PCA) was generated by the Origin 2023 procedure (Origin Lab Corp., Northampton, MA, USA) to visualize the interrelationships among the parameters regarding the antioxidant system and the N-assimilation pathway investigated in this study.

## 3 Results

### 3.1 The rapeseed plant growth and morphology

**Figure 2:**
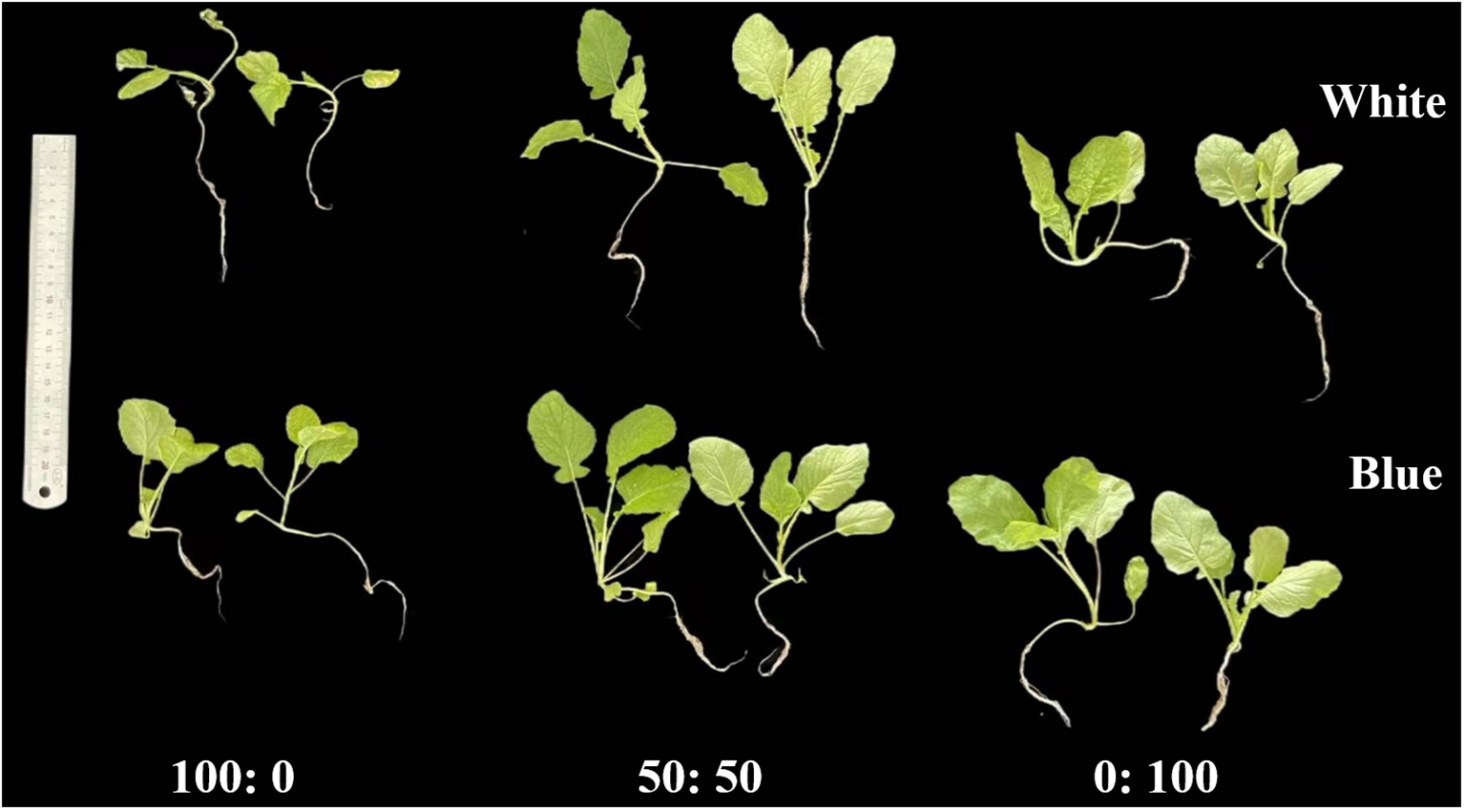
Morphological changes of rapeseed under white or blue light in response to three NH_4_^+^:NO_3_^−^ ratios after several weeks of treatment. Ratios of 100:0, 50:50, and 0:100 NH_4_^+^:NO_3_^−^ are shown from left to right, with each treatment applied to two rapeseed plant replicates of similar size.

The rapeseed plants showed significantly different morphological appearances regarding shoot length, leaf area, and the root system (**Figure 2**). Setting light quality aside, it is clear that rapeseed plants treated with 50:50 NH_4_^+^:NO_3_^−^ showed more vigorous and healthy growth compared with those cultured in the other two regimes. In contrast, solely NH_4_^+^-cultured plants displayed severely restricted growth and development, and solely NO_3_^−^-cultured plants exhibited better growth than those grown in the 100:0 NH_4_^+^:NO_3_^−^.

However, blue light imparted greater growth ability compared with plants cultured under a white environment, regardless of the NH_4_^+^:NO_3_^−^ ratio (**Figure 2**). In particular, for rapeseed plants solely supplied with 100% NH_4_^+^, blue light notably mitigated the reduced growth compared with that observed under white light (**Figure 2**).

### 3.2 The plant growth parameters

**Figure 3:**
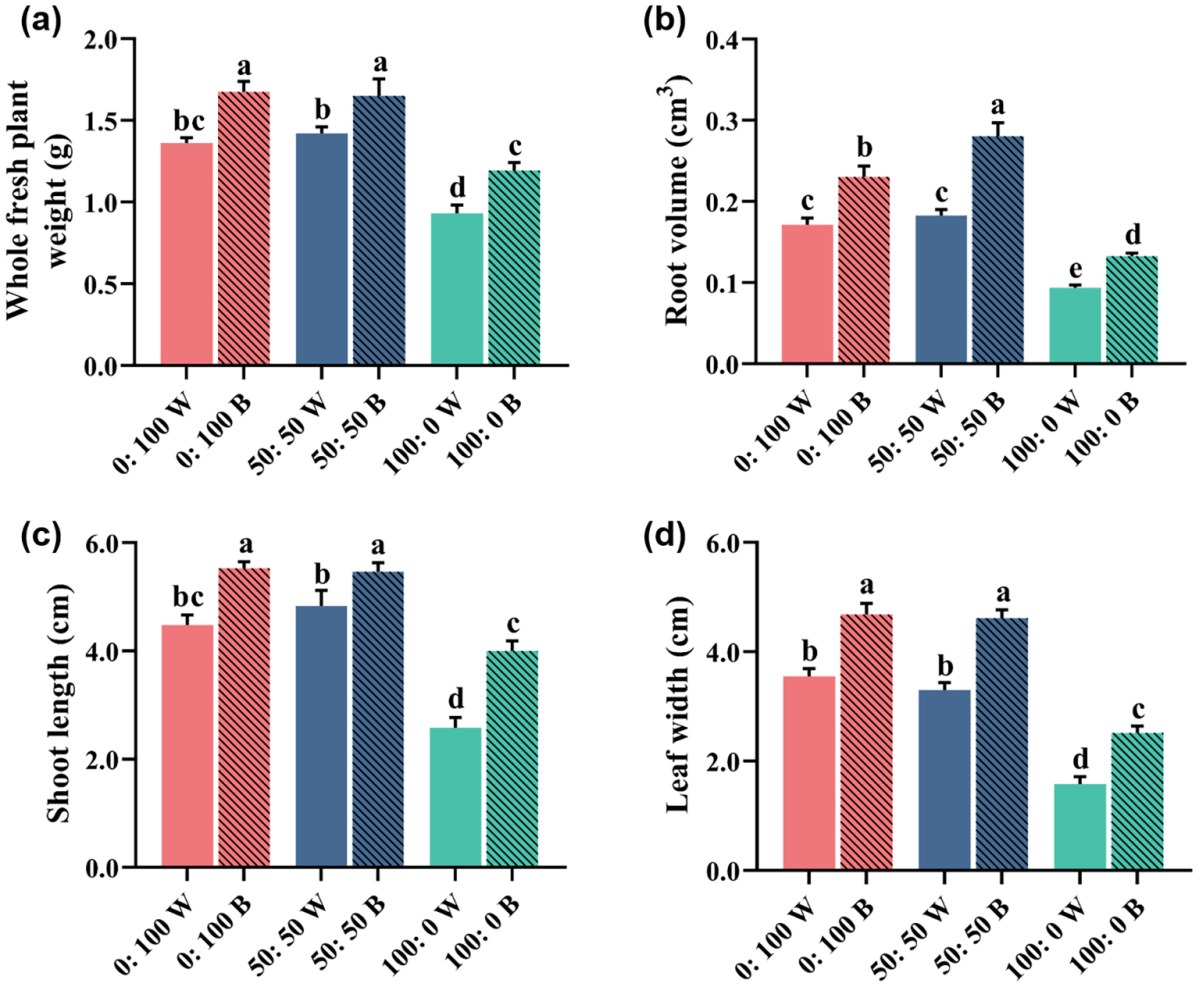
Rapeseed growth parameters: **(a)** whole plant fresh weight, **(b)** root volume, **(c)** shoot length, and **(d)** leaf width as affected by three NH_4_^+^:NO_3_^−^ ratios under white or blue light. Data are means ± SE from six biological replicates (*n* = 6). Significant differences among treatments were determined by Duncan’s multiple comparison range test at *p* = 0.05 (one-way ANOVA) and are denoted by different lowercase letters above the bars.

Indeed, the recorded responses of plant growth and morphology to different NH_4_^+^:NO_3_^−^ treatments and light qualities were further supported by the investigated growth parameters (**Figure 3**; **Table 2**).

**Table 2:** Rapeseed dry weight, stem diameter, tap root length, root surface area, total root length, and leaf length in response to three NH_4_^+^:NO_3_^−^ ratios under white or blue light.

Treatment (NH_4_^+^:NO_3_^−^)	Dry weight (mg)	Stem diameter (mm)	Tap root length (cm)	Root surface area (cm^2^)	Total root length (cm)	Leaf length (cm)
0:100 W	89.3^a^ b^b^	1.5 b	6.1 bc	22.9 b	170.6 b	4.4 b
0:100 B	173.0 a	2.0 a	8.8 a	38.2 a	229.7 a	5.4 a
50:50 W	89.8 b	1.4 b	6.7 b	25.4 b	171.1 b	4.6 b
50:50 B	161.0 a	2.1 a	8.2 a	38.0 a	246.6 a	5.7 a
100:0 W	38.7 d	1.0 d	4.1 d	8.7 d	71.7 c	2.1 d
100:0 B	66.3 c	1.2 c	5.6 c	15.1 c	89.6 c	3.0 c

a Data in the table are average ± SE generated from six biological replicates (*n* = 6).

b Data accompanied by different lowercase letters indicate significant differences at *p* = 0.05 among different treatments.

Across **Figure 3**, rapeseed plants grown in the 0:100 and 50:50 NH_4_^+^:NO_3_^−^ regimes displayed similar trends, while 100% NH_4_^+^ nutrition conferred dramatically reduced growth, regardless of light quality. More importantly, compared with rapeseed plants grown under white light, blue light-treated plants significantly improved these traits by varying degrees, especially for the solely NH_4_^+^-treated plants. For example, for plants grown in the 100:0 NH_4_^+^:NO_3_^−^ regime, blue light significantly increased shoot length by 60% relative to those cultivated under white light (**Figure 3c**).

Concomitantly, other important growth parameters such as dry weight, stem diameter, tap root length, root surface area, total root length, and leaf length also showed similar responses to the different NH_4_^+^:NO_3_^−^ ratios and light qualities (**Table 2**). It is still worth noting that 100% NH_4_^+^ nutrition caused significant decreases in plant growth ability, whereas this phenomenon was markedly alleviated when plants were cultured under blue light.

### 3.3 Ammonium toxicity in solely NH_4_^+^-cultured rapeseed plants

**Figure 4:**
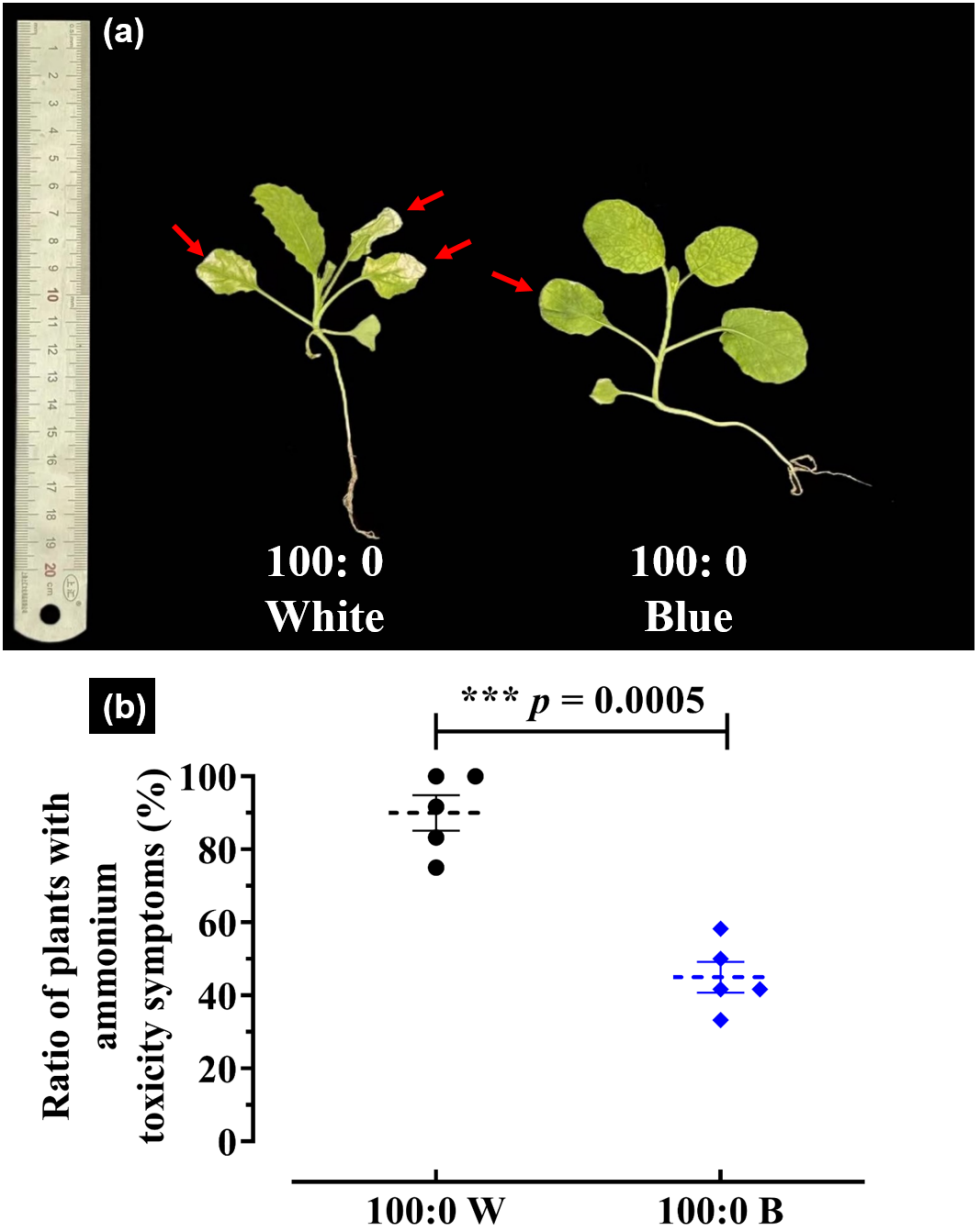
NH_4_^+^ toxicity-related parameters in rapeseed plants supplied solely with NH_4_^+^: **(a)** plant growth status comparison and **(b)** ratio of plants with NH_4_^+^ toxicity symptoms (%) under white or blue light. Red arrows in **(a)** indicate typical NH_4_^+^ toxicity symptoms in the foliage. Data in **(b)** are means ± SE from six biological replicates (*n* = 6). The significant differences between white light and blue light were determined according to a two-tailed Student’s *t*-test.

Notably, the rapeseed plants treated with the 100:0 NH_4_^+^:NO_3_^−^ solution eventually developed NH_4_^+^ toxicity symptoms, regardless of light quality. This phenomenon was characterized by chlorosis and visible foliage necrosis accompanied by burned tips, stunted roots, and inhibited growth (**Figure 4a**).

However, blue light-cultivated rapeseed plants significantly ameliorated the degree of NH_4_^+^ toxicity and improved plant growth. Indeed, the appearance ratio of plants with NH_4_^+^ toxicity symptoms under blue light drastically declined from 91.7% to 41.7%, compared with that under white light conditions (**Figure 4b**).

### 3.4 Photosynthetic ability

**Figure 5:**
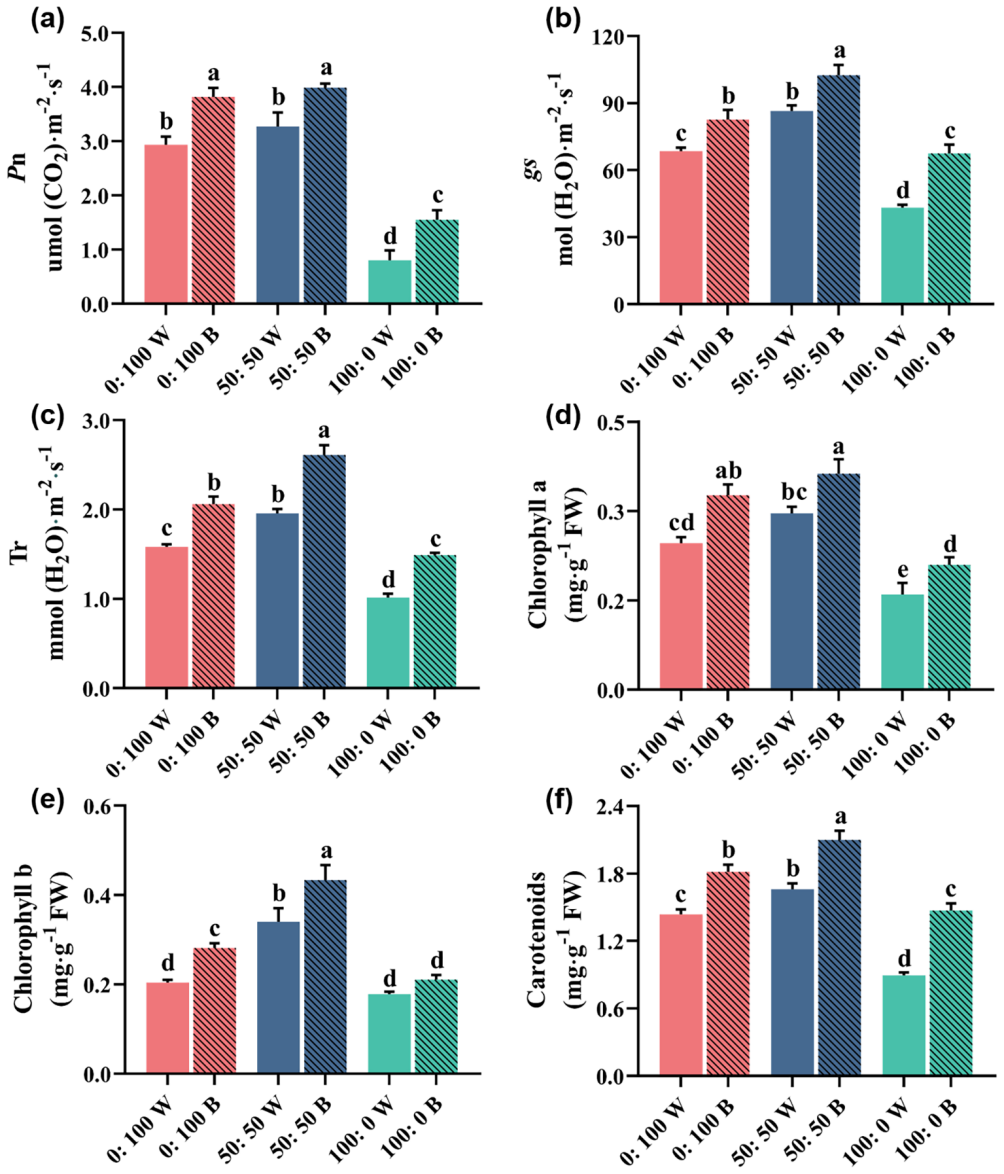
Photosynthetic ability-related parameters: **(a)** net photosynthesis rate (Pn), **(b)** stomatal conductance (*g*_s_), **(c)** transpiration rates (Tr), **(d)** chlorophyll *a*, **(e)** chlorophyll *b*, and **(f)** carotenoids as affected by three NH_4_^+^:NO_3_^−^ ratios and light quality. Data are means ± SE from six biological replicates (*n* = 6). Significant differences among treatments were denoted by different lowercase letters (one-way ANOVA following Duncan’s multiple comparison range test at *p* = 0.05).

The photosynthesis of rapeseed plants was also distinctly altered in response to high NH_4_^+^ or NO_3_^−^ supply under white light or blue light conditions. Thus, the photosynthetic ability regarding Pn, *g*_s_, Tr, chlorophyll *a*, chlorophyll *b*, and carotenoids was assessed (**Figure 5**).

Specifically, it is worth noting that plants treated with 100% NH_4_^+^ nutrition significantly decreased all the investigated parameters, regardless of light quality. Overall, irrespective of the light conditions considered, plants grown in the 0:100 NH_4_^+^:NO_3_^−^ regime showed similar trends to those in the 50:50 NH_4_^+^:NO_3_^−^ regime, with the exception of Pn (**Figure 5a**).

Blue light-treated plants significantly improved the photosynthetic ability compared with plants grown under white light, regardless of the NH_4_^+^:NO_3_^−^ ratios (**Figure 5**). In particular, the disturbed photosynthetic ability of 100% NH_4_^+^-supplied plants under white light was notably mitigated when treated with blue light.

### 3.5 Antioxidant enzyme activity and ROS

The oxidative protective system is triggered when plants are subjected to external stresses. In this regard, antioxidant enzyme activities increase to enhance antioxidant capacity and reduce ROS accumulation.

**Figure 6:**
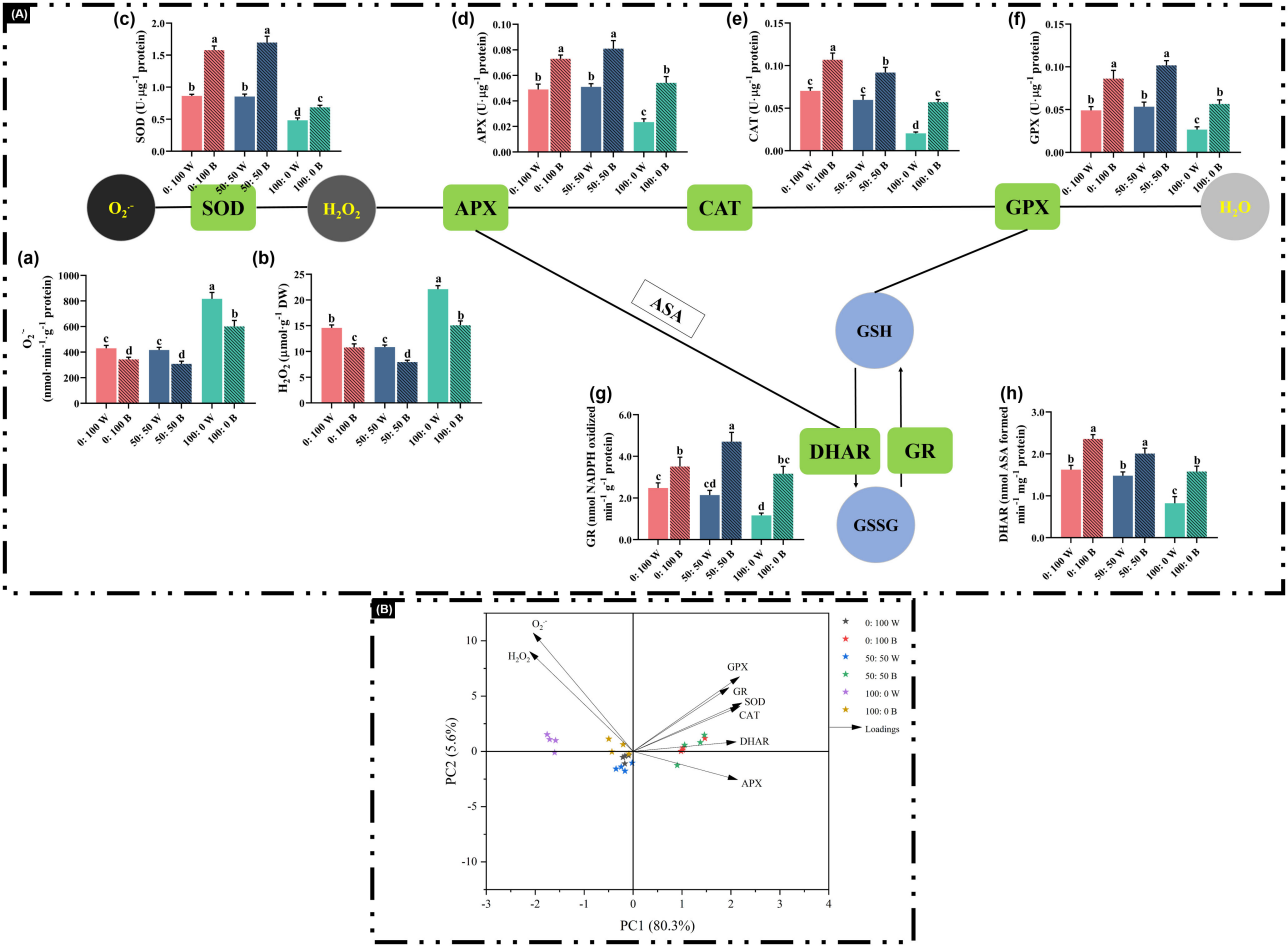
Analysis of the antioxidant defense system: **(A)** ROS content ((a) O_2_.^−^ and (b) H_2_O_2_) major antioxidant enzyme activities ((c) SOD, (d) APX, (e) CAT, (f) GPX, (g) DHAR, (h) GR) and **(B)** multivariate data analysis by PCA. Data are means ± SE from no less than four biological replicates (*n* ≥ 4). Significant differences among treatments were denoted by different lowercase letters (one-way ANOVA following Duncan’s multiple comparison range test at *p* = 0.05).

Indeed, plants treated solely with NH_4_^+^ showed significantly increased accumulations of O_2_.^−^ and H_2_O_2_ compared with those cultivated with 100% NO_3_^−^ or a mixed NH_4_^+^ and NO_3_^−^ (**Figures 6A** [a, b]). The antioxidant enzyme activities, however, did not improve as the external NH_4_^+^ supply increased from 50% to 100%. Notably, compared with plants cultured under white light, blue light-treated plants markedly increased major antioxidant enzyme activities and consequently reduced ROS accumulations (**Figure 6A**).

Moreover, the impacts of three NH_4_^+^:NO_3_^−^ solutions under white light or blue light on antioxidant enzymes and oxidative damage, along with the relationships among all treatments, were visualized using PCA. The PCA results along the first two principal dimensions (“PC1” = 80.3%, “PC2” = 5.6%) explained a total data variability of 85.9%. Overall, blue light-treated plants were mainly distributed on the right side of PC1, whereas white light-treated plants were primarily located on the left side of PC1 (**Figure 6B**). Additionally, blue light-treated plants exhibited higher antioxidant enzyme activities and lower ROS concentrations.

### 3.6 Key enzymes, activities, and chemical contents in the N-assimilation pathway

To investigate whether blue light affects the major N-assimilation enzymes and key chemical contents in the N-assimilation pathway during NH_4_^+^ toxicity alleviation, the activities of NR, NIR, GS, GOGAT, GDH, as well as the concentrations of NO_3_^−^, NO_2_^−^, and NH_4_^+,^ were determined.

**Figure 7:**
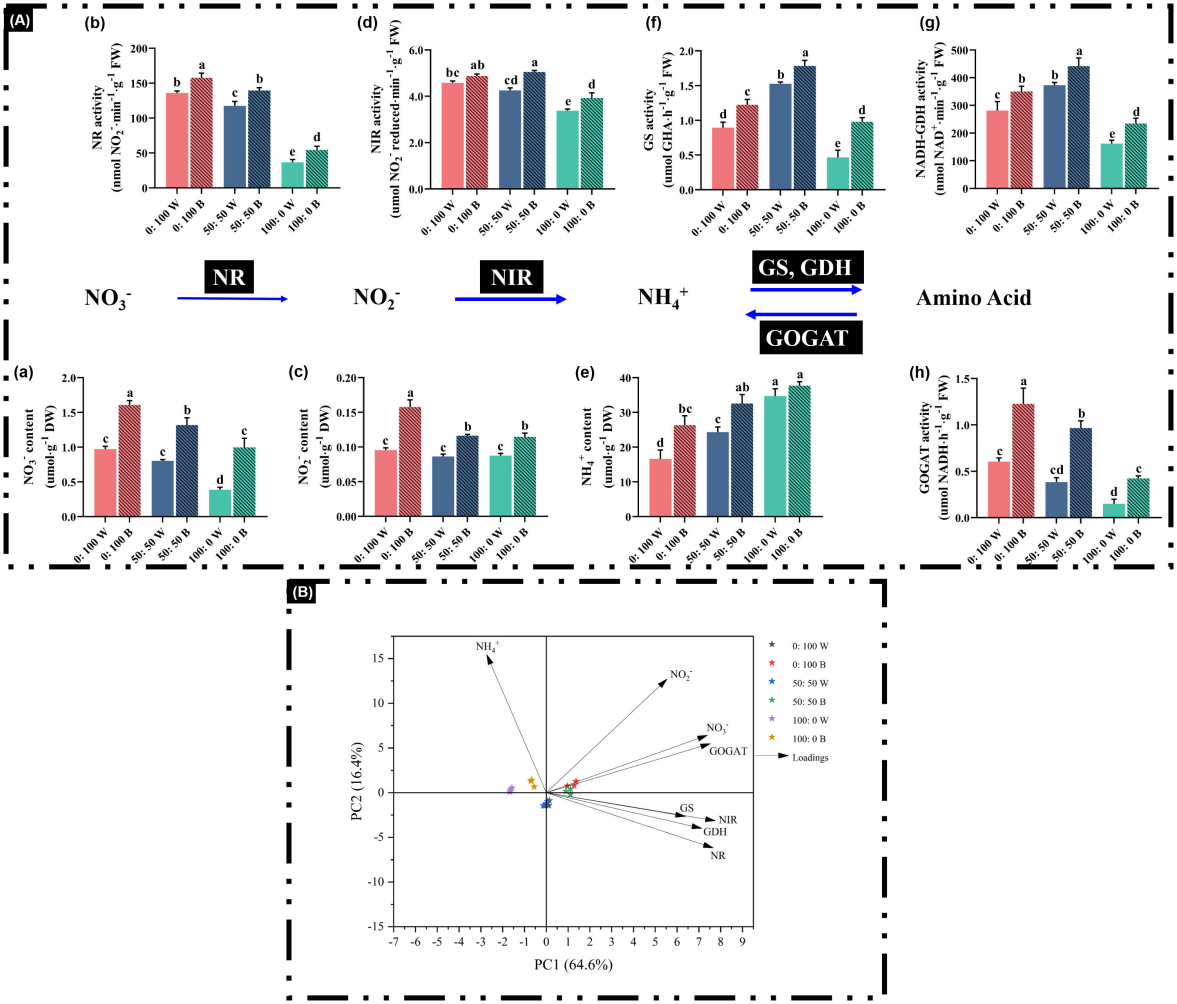
Analysis of main chemicals: **(A)** (a) NO_3_^−^ content, (c) NO_2_^−^ content, and (e) NH_4_^+^ content and major enzymes: **(A)** (b) NR activity, (d) NIR activity, (f) GS activity, (g) NADH-GDH activity, and (h) GOGAT activity in the N assimilation pathway, as well as **(B)** multivariate data analysis by PCA. Data are means ± SE from no less than four biological replicates (*n* ≥ 4). Significant differences among treatments were denoted by different lowercase letters (one-way ANOVA following Duncan’s multiple comparison range test at *p* = 0.05).

It is noteworthy that a high supply of NO_3_^−^ or NH_4_^+^ results in correspondingly high levels of free NO_3_^−^ or NH_4_^+^ in plants, respectively, regardless of light quality (**Figures 7A** [a, e]). Similarly, GS and GDH were significantly increased when external NH_4_^+^ nutrition was elevated from 0% to 50%, regardless of light quality (**Figures 7A** [f, g]). Conversely, the GOGAT activity gradually decreased in response to declining NH_4_^+^ nutrition supply (**Figures 7A** [h]).

However, the blue light-treated plants not only dramatically increased the activities of enzymes in the N-assimilation pathway, particularly GS, GDH, and GOGAT (**Figures 7A**, [f–h]), but also notably increased the contents of free chemicals, such as NO_3_^−^ (**Figures 7A** [a]). Importantly, 100% NH_4_^+^-treated plants under blue light showed marked enhancement of enzyme activities and chemical contents, except for free NH_4_^+^ concentrations (**Figure 7A**), compared to plants cultivated under white light. For instance, GS, GDH, and GOGAT activities in plants under the 100:0 NH_4_^+^:NO_3_^−^ regime with blue light were significantly increased by 1.13-fold, 44.8%, and 1.83-fold, respectively (**Figures 7A**[f–h]).

All investigated parameters were analyzed through PCA to visualize how the NH_4_^+^:NO_3_^−^ ratios and light quality affected the N-assimilation pathway. The first two principal components explained 81% (PC1 = 64.6%, PC2 = 16.4%) of the total variance (**Figure 7B**). On average, plants treated with blue light were mainly separated along PC2, while those treated with white light were located in the opposite direction, illustrating the contrasting traits between blue and white light treatments.

## 4 Discussion

A high or exclusive supply of NH_4_^+^ inevitably inhibits plant growth and development. In our trials, numerous morphological and physiological rapeseed traits were significantly altered by a high external NH_4_^+^ concentration, as shown by decreased plant weight, reduced plant shoot and leaf, and declined root-related parameters, including root volume and total root length (**Figure 3**; **Table 2**). The rapeseed plants in this study also developed NH_4_^+^ toxicity symptoms, characterized by severely restricted growth and development, chlorosis, necrosis, and stunted roots (**Figure 4a**). These detrimental effects of NH_4_^+^ toxicity are consistent with observations in other NH_4_^+^-sensitive plant species, such as basil (Song et al., 2024a), cabbage (Song et al., 2022a), and beans (Guo et al., 2002). These findings indicate that rapeseed is highly sensitive to high NH_4_^+^ supply and can be classified as an NH_4_^+^-sensitive plant.

Data to date on the alleviation of NH_4_^+^ toxicity in plants by blue light treatment are very limited, especially for NH_4_^+^-sensitive plant species (Bittsánszky et al., 2015; Esteban et al., 2016; Shilpha et al., 2023). However, blue light-induced alleviation of abiotic stresses has been reported in many plant species. For instance, blue light-treated pepper showed improved photosynthesis under UV light stress (Hoffmann et al., 2015), and it was suggested that drought resistance was increased through blue light regulation in melo (Li X et al., 2024). Several pioneering studies revealed that blue light application significantly promoted plant growth and development, ranging from agricultural crops to horticultural flowers or vegetables (Zheng and Van Labeke, 2017; Jin et al., 2023; Li et al., 2021). Our data also showed that rapeseed growth was notably improved under blue light, regardless of the NH_4_^+^:NO_3_^−^ ratios (**Figures 2**, **3**; **Table 2**), further demonstrating the beneficial effects of blue light on rapeseed plants.

Moreover, although growth parameters were severely inhibited in rapeseed plants grown under a 100% NH_4_^+^ regime with white light, blue light-spiked plants showed a significant alleviation of NH_4_^+^ toxicity, reducing these growth limitations (**Figures 2**, **3**; **Table 2**). This finding further confirms the alleviatory role of blue light under this abiotic stress and contributes to sustainable crop production and higher productivity quality. In solely NH_4_^+^-cultured rapeseed plants, blue light treatment significantly reduced the occurrence of NH_4_^+^ toxicity (%) from 91.7% to 41.7%, compared to plants grown under white light (**Figure 4**). These results also demonstrated that NH_4_^+^ toxicity can be notably lessened in rapeseed after blue light treatment.

The photosynthesis of rapeseed plants was also severely limited when 100% NH_4_^+^ nutrition was applied, regardless of light quality, as indicated by the significantly reduced Pn, *g*_s_, Tr, chlorophylls, and carotenoids (**Figure 5**). This phenomenon was likely due to abiotic stresses impairing the performance of photosystems, chlorophyll biosynthesis, gas exchange parameters, and electron transport mechanisms (Sharma et al., 2020). In addition, overproduction of ROS caused oxidative damage, interfered with electron transport mechanisms, and even injured the chloroplast (Sharma et al., 2020; Guo et al., 2016). These damages to photosynthesis caused by high NH_4_^+^ nutrition are consistent with many previous reports, including those on cabbage (Song et al., 2021), salvia (Song et al., 2022b), and basil (Song et al., 2024b). As expected, the inhibition of photosynthesis by 100% NH_4_^+^ supply was markedly mitigated when rapeseed plants were grown under blue light (**Figure 5**). Blue light significantly improved the investigated photosynthesis-related parameters, further confirming its ability to alleviate NH_4_^+^ toxicity and enhance photosynthetic capacity. Hogewoning et al. (2010) reported that leaf photosynthesis increased quantitatively when the proportion of blue light reached a “qualitative” or “threshold”. Blue light has also been shown to promote stomatal opening more effectively than other wavelengths, resulting in higher photosynthetic rates and providing additional morphogenetic benefits (Wang et al., 2015). Overall, blue light treatment effectively attenuated the decreases in Pn, *g*_s_, Tr, and chlorophyll content, thereby maintaining photosynthetic capacity.

In this study, the blue light treatment was not 100% but was mixed with other light qualities (**Figure 1b**). Compared with white LED light, we increased the proportion of blue light and reduced other light qualities to achieve the blue light treatment. Previous studies found that physiological disorders in cucumber were eliminated after adding a small amount of blue light (Hogewoning et al., 2010). Hoffmann et al. (2015) demonstrated that higher amounts of blue light triggered better photosynthetic performance and greater pigment accumulation. Our data are consistent with these findings, showing that increasing the proportion of blue light promotes photosynthetic characteristics.

Usually, a dynamic equilibrium between the accumulation and scavenging of ROS is well maintained in plants under a normal environment (Gong et al., 2008; Song et al., 2022a). Meanwhile, the steady-state level of ROS is regulated in association with the antioxidant defense systems, mainly through stimulated antioxidative enzymes (Ali et al., 2019). For instance, as a prime candidate, SOD participates not only in the conversion of O_2_.^−^ into H_2_O_2_ but also in the subsequent decomposition of H_2_O_2_ to H_2_O via APX, CAT, and GPX (Gong et al., 2008; Ali et al., 2019; Song et al., 2022a). Nevertheless, NH_4_^+^ toxicity causes imbalances and disturbances in redox signaling, as evidenced by increased accumulations of O_2_.^−^ and H_2_O_2_ (**Figures 6A** [a, b]), suggesting that substantial externally spiked NH_4_^+^ obstructs the dynamic balance of ROS between production and elimination in rapeseed plants. In addition, antioxidant enzyme activities failed to increase in response to progressively higher NH_4_^+^ supply from 50% to 100%, regardless of light quality, further confirming that rapeseed plants in this experiment were extremely sensitive to high NH_4_^+^.

Many studies have explored the impacts of blue light on the regulation of the oxidative defense system, showing that blue light can induce tolerance against abiotic stresses (Sebastian and Prasad, 2014; Pech et al., 2024). In our investigation, blue light stimulated higher activities of antioxidant enzymes compared with those grown in white light, thereby neutralizing excessive O_2_.^−^ and H_2_O_2_ (**Figure 6A**). This enhancement of antioxidant enzymes provided a protective role of blue light on the cell membrane (Shao et al., 2020). These findings are in line with previous publications showing that blue light treatment can enhance the antioxidant defense system and improve plant quality (Xu et al., 2014; Manivannan et al., 2015). NH_4_^+^ toxicity-caused excessive oxidative stress in rapeseed plants was clearly alleviated when plants were cultured under blue light conditions. Phototropins (PHOT) are one of three classes of receptors that modulate blue light responses. They are cytosolic and plasma membrane-associated photoreceptors that play important roles in adaptation to oxidative stresses (Chibani et al., 2025). Cryptochromes (Crys) are flavin-binding blue light receptors that regulate ROS generation. They absorbed blue light and act as key regulators in response to multiple abiotic stresses (El-Esawi et al., 2017). The mitigation of NH_4_^+^ toxicity-caused excessive oxidative stress by blue light in this study may be partially attributed to these blue light receptors.

In higher plants, the N-use pathway is conserved and involved in many biological and biochemical processes (i.e., uptake, assimilation, and translocation) (Masclaux-Daubresse et al., 2010). Plants were not to assimilate NO_3_^−^ directly, but reduce it to NO_2_^−^ via NR. NO_2_^−^ is further converted to NH_4_^+^ via NIR, and NH_4_^+^ is finally incorporated, catalyzed, and assimilated through the GS/GOGAT pathway or alternatively taken up by GDH (**Figure 7A**) (Cruz et al., 2006; Bittsánszky et al., 2015; Song et al., 2021). A positive correlation between NR activity and free NO_3_^−^ content was observed, and similarly, high NH_4_^+^ supply led to high free NH_4_^+^ accumulation in plants (**Figures 7A** [a, b, e]), consistent with previous reports (Cruz et al., 2006; Horchani et al., 2010; Liu et al., 2017; Song et al., 2021). Unlike NR, NIR activity decreased significantly only when external NH_4_^+^ supply increased rapidly from 50% to 100%, regardless of light quality, in agreement with Song et al. (2021). Regulation of NR and NIR under high NO_3_^−^ or NH_4_^+^ supply could inevitably affect the activities of downstream GS, GOGAT, and GDH (Song et al., 2021). We observed that the enhancement of GS and NADH-GDH under external NH_4_^+^ supply increased from 0% to 50%, illustrating a regulatory mechanism in rapeseed in response to high environmental NH_4_^+^. By contrast, an opposite regulatory pattern of GOGAT compared with GS and NADH-GDH was observed (**Figures 7A** [h]).

Most importantly, blue light treatments on rapeseed significantly improved the N assimilation pathway-related enzymes and chemicals mentioned above compared with plants cultured under white light (**Figure 7A**). Numerous pioneering studies have shown that NH_4_^+^ tolerance is improved by progressive and sustained upregulations of NH_4_^+^ assimilation enzyme activities (Horchani et al., 2010; Esteban et al., 2016; Song et al., 2021, 2022, 2022, 2022). Blue light-treated rapeseed plants exhibited enhanced N assimilation enzymes and chemicals in the N assimilation pathway, suggesting positive effects conferred by blue light due to better growth performance under a blue light environment (**Figures 2**, **3**; **Table 2**). Notably, blue light-treated plants showed increased activities of NR, NIR, GS, GOGAT, and NADH-GDH and markedly reduced NH_4_^+^ toxicity in plants treated with 100% NH_4_^+^ (**Figures 4a**, **7A**). Additionally, boosting or maintaining high GS and GDH levels is considered an important strategy for NH_4_^+^ tolerance; in other words, GS and GDH can underpin NH_4_^+^ tolerance to some extent in plant species (Song et al., 2021, 2022; Xian et al., 2020). As a consequence, the improved NR/NIR route, GS/GOGAT cycle, and GDH/GOGAT cycle contribute to enhanced NUE and NH_4_^+^ tolerance, thereby reducing NH_4_^+^ toxicity (Esteban et al., 2016; Peng et al., 2023). Blue light-treated plants also showed higher free contents of NO_3_^−^, NO_2_^−^, and NH_4_^+^ compared with those cultured under white light (**Figure 7A**), indicating that a higher uptake rate of these chemicals was associated with improved NUE under blue light. Accordingly, NH_4_^+^ toxicity in rapeseed plants can be alleviated by blue light treatment through improvement of the N assimilation pathway, including GS and GDH activities.

Interestingly, a similar study by Sakuraba and Yanagisawa (2018) revealed that phytochromes (Phys) and Crys perceive blue light signals and play an important role in nitrate assimilation and the regulation of NR activity. In cabbage, certain phytochrome*-*related genes (*PHYA*, *PHYC*, and *PHYE*) and cryptochrome-related genes (*CRY2a* and *CRY2b*) were significantly upregulated under a blue light regime compared to white light, resulting in higher concentrations of NR, NIR, and GS (Fan et al., 2022). These blue light receptors may also participate in the N metabolism pathway in plants.

## 5 Conclusion

In summary, this work demonstrates that NH_4_^+^ toxicity in rapeseed plants is significantly alleviated under blue LED light treatment. This amelioration effect in NH_4_^+^-stressed rapeseed plants involves multiple aspects, mainly including ameliorated growth, enhanced photosynthesis, strengthened antioxidative machinery, and a more efficient N assimilation pathway. These findings suggest that blue light is highly effective in reinforcing the NH_4_^+^ tolerance and promoting the growth and quality of rapeseed plants.

The current endeavor not only provides new avenues for applying blue light to enhance NH_4_^+^ tolerance but also offers considerable practical value in PFALs for increasing rapeseed productivity. Concomitantly, plant yield and safety could be ensured under this lighting regime, and a precise manipulation of blue light in practical agriculture could alter plant morphological plasticity, thereby promoting desirable commercial value. Further study of the alleviatory role of blue light in NH_4_^+^ toxicity at the molecular level is warranted.

## Data Availability

The raw data supporting the conclusions of this article will be made available by the authors, without undue reservation.
